# *NOTCH3* Variants and Genotype-Phenotype Features in Chinese CADASIL Patients

**DOI:** 10.3389/fgene.2021.705284

**Published:** 2021-07-15

**Authors:** Yacen Hu, Qiying Sun, Yafang Zhou, Fang Yi, Haiyun Tang, Lingyan Yao, Yun Tian, Nina Xie, Mengchuan Luo, Zhiqin Wang, Xinxin Liao, Hongwei Xu, Lin Zhou

**Affiliations:** ^1^Department of Geriatric Neurology, Xiangya Hospital, Central South University, Changsha, China; ^2^National Clinical Research Center for Geriatric Disorders, Xiangya Hospital, Central South University, Changsha, China; ^3^Department of Radiology, Xiangya Hospital, Central South University, Changsha, China

**Keywords:** CADASIL, *Notch3*, untypical variant, cysteine-sparing variant, genotype-phenotype

## Abstract

Cerebral autosomal dominant arteriopathy with subcortical infarcts and leukoencephalopathy (CADASIL) is a cerebral small vessel disease caused by mutations in the *NOTCH3* gene. Archetypal disease-causing mutations are cysteine-affecting variants within the 34 epidermal growth factor-like repeat (EGFr) region of the Notch3 extracellular subunit. Cysteine-sparing variants and variants outside the EGFr coding region associated with CADASIL phenotype have been reported. However, the linkage between untypical variants and CADASIL is unclear. In this study, we investigated the spectrum of *NOTCH3* variants in a cohort of 38 probands from unrelated families diagnosed as CADASIL. All coding exons of the *NOTCH3* gene were analyzed, and clinical data were retrospectively studied. We identified 23 different *NOTCH3* variants including 14 cysteine-affecting pathogenic variants, five cysteine-sparing pathogenic variants, two reported cysteine-sparing variants of unknown significance (VUS), and two novel VUS outside EGFr region. In retrospective studies of clinical data, we found that patients carrying cysteine-sparing pathogenic variants showed later symptom onset (51.36 ± 7.06 vs. 44.96 ± 8.82, *p* = 0.023) and milder temporal lobe involvement (1.50 ± 1.74 vs. 3.11 ± 2.32, *p* = 0.027) than patients carrying cysteine-affecting pathogenic variants. Our findings suggested that untypical variants comprise a significant part of *NOTCH3* variants and may be associated with a distinctive phenotype.

## Introduction

Cerebral autosomal dominant arteriopathy with subcortical infarcts and leukoencephalopathy (CADASIL, OMIM NO.125310) is a cerebral small vessel disease caused by mutations in the *NOTCH3* gene (Joutel et al., 1996). Clinical manifestation includes migraine, recurrent cerebrovascular events, psychiatric disturbance, and cognitive impairment that eventually lead to dementia and disability. Magnetic resonance imaging (MRI) is characterized by multiple lacunes and extensive white matter hyperintensity (WMH), especially in the anterior temporal lobe and external capsule (Chabriat et al., 2009). Deposition of Notch3 extracellular domain or granular osmiophilic material (GOM) in small arteries is a pathological hallmark of CADASIL (Joutel et al., 2000). However, due to the prominent heterogeneity of clinical manifestation, genetic testing remains the gold standard for diagnosis.

The *NOTCH3* gene contains 33 exons encoding the Notch3 protein, a single-pass transmembrane receptor of 2,321 amino acids. Notch3 contains a large extracellular domain (ECD) that consists of 34 epidermal growth factor-like repeats (EGFr), a transmembrane domain (TMD), and an intracellular domain (ICD) (Wang et al., 2008). To date, more than 300 *NOTCH3* mutations have been reported, and most of the pathogenic mutations reside in exons 2–24 coding for EGFr 1–34 in the ECD of the Notch3 protein. Most mutations are missense mutations that change the number of cysteines in EGFr, leading to misfolding of the receptor and aggregation of the extracellular domain (Rutten et al., 2014).

Thus far, the vast majority of pathogenic mutations were found within the EGFr region of the Notch3 extracellular domain, encoded by exons 2–24 (Rutten et al., 2014). Some other studies show that mutations after exon 24 may not be associated with CADASIL phenotype rather than other diseases, such as infantile myofibromatosis (Martignetti et al., 2013) and lateral meningocele syndrome (Gripp et al., 2015). Therefore, most previous studies in CADASIL patients only analyzed the *NOTCH3* gene by sequencing exons 2–24. However, several studies reported mutations outside the EGFr coding region (exons 25–33) to be possible causal of CADASIL (Jung et al., 1995; Joutel et al., 1996; Fouillade et al., 2008; Bersano et al., 2012; Hung et al., 2018). On the other hand, *NOTCH3* mutations are not detected in all biopsy-determined CADASIL patients by sequencing the EGFr encoding region (Peters et al., 2005), suggesting that variants outside the EFGr encoding region require further investigation.

In this study, we investigated the spectrum of *NOTCH3* variants in Chinese CADASIL patients by sequencing all coding exons of *NOTCH3*. We found that untypical variants comprise a significant part of *NOTCH3* variants and may be associated with a distinctive phenotype.

## Materials and Methods

### Patients

Fifty-four probands of unrelated families clinically diagnosed as CADASIL were collected by National Clinical Research Center for Geriatric Disorders (Xiangya) between 2014 and 2020. All patients were of Chinese Han ethnicity and came from central-south region of mainland China. They were diagnosed as CADASIL based on the following criteria: (1) positive family history of stroke, dementia, or migraine; (2) symmetrical MRI abnormalities suggestive of vascular WMH; and (3) presence of more than one typical symptom: recurrent stroke especially at relatively early age, migraine, cognitive impairment, or psychiatric symptoms. Written informed consent was obtained from each subject, and the study protocol was approved by the institutional review boards of Xiangya Hospital, Central South University.

### Collecting Clinical Information

Data on the background (gender, age at onset, age at assessment, family history, and vascular risk factors), clinical symptoms (migraine, ischemia/hemorrhagic stroke, cognitive impairment, and psychiatric symptoms), skin biopsy results, and MRI findings from proband and family members were collected. The severity of WMH was scored by the modified Schelten’s scale (Scheltens et al., 1993). The score was assigned according to the following: 0 = absent; 1 = up to five lesions < 3 mm diameter; 2 = six or more lesions < 3 mm diameter; 3 = up to five lesions 4–10 mm in diameter; 4 = six or more lesions 4–10 mm in diameter; and 5 = one or more lesions > 10 mm diameters; 6 = confluent hyperintensities. Cerebral microbleeds (CMBs) were counted in susceptibility weighted imaging (SWI) sequences. CMB burden were categorized according to the following: 0 = absent; 1 = up to five CMBs; and 2 = six or more CMBs. The WMH rating and CMB categorizing were performed by one experienced vascular neurologist who was blind to all clinical data. Age at onset of migraine was not used as age at onset of CADASIL because occurrence of migraine was markedly earlier than other symptoms. Risk factors include hypertension, smoking, diabetes, and hyperlipidemia.

### Genetic Analysis

Genomic DNA from patients and controls was extracted according to the manufacturer’s standard procedure using the QIAamp DNA Blood Midi Kit (Qiagen, Hilden, Germany). Polymerase chain reaction (PCR) was performed with primers (comprising intron–exon boundaries) specific for 33 exons of the *NOTCH3* gene. Following purification of PCR products, sequencing was performed using the automated sequencer ABI 3730 (Applied Biosystems, Foster City, CA, United States). Sequencing in the opposite direction was performed whenever an abnormal sequence was found. For patients with variants outside EGFr region, additional genetic analyses were performed to exclude mutations in several other genes known to be causal of cerebral small-vessel disease or vascular dementia (*HTRA1*, *TREX1*, *COL4A1*, *COL4A2*, *GLA*, *APP*, and *ITM2B*) by targeted next-generation sequencing (data unpublished). In addition, 400 age- and sex-matched Chinese controls were screened for variants outside EGFr region in the *NOTCH3* gene to determine the allele frequency in the Chinese population.

### Bioinformatic Analyses of Untypical Variants

Four functional prediction software programs were used to predict the effect of untypical variants on protein structure, function, and sequence conservation (SIFT, PolyPhen-2, mutation taster, and GERP^++^). Using SIFT, scores ranging from 0 to 1 are obtained to represent the normalized probability that a particular amino acid substitution will be tolerated. SIFT predicts that substitutions with scores less than 0.05 are damaging (Ng and Henikoff, 2001). In PolyPhen-2, a variant is classified as “probably damaging” if it has a probabilistic score greater than 0.85, and “possibly damaging” if it has a score greater than 0.15; the remaining variants are classified as benign (Adzhubei et al., 2010). The Mutation taster probability value ranging from 0 to 1 represents the probability of the prediction, in which a value close to 1 indicates a high security of the prediction (Schwarz et al., 2014). In GERP^++^, a variant is classified as “conserved” if it has a probabilistic score greater than 2.00 (Davydov et al., 2010). We used the Genome Aggregation Database (GnomAD)^1^ to establish the minor allele frequency (MAF) of novel variants.

### Statistical Analyses

Statistical analyses were performed using SPSS version 20 software (SPSS Inc., Chicago, IL). We compared clinical features in patients by Mantel–Haenszel chi-square statistic test or Fisher’s exact tests for categorical variables and unpaired *t*-test for continuous variables. Values of *p* < 0.05 were considered statistically significant.

## Results

### Demographic and Clinical Feature Description

*NOTCH3* variants were detected in 38 out of 54 proband patients. The demographic and clinical features of all proband patients are summarized in Table 1. For patients carrying *NOTCH3* variants, average age of symptom onset was 47.18 ± 9.51 years (range 24–72 years). Throughout the disease course, ischemic stroke/transient ischemic attack (TIA) was the most common symptom (32/38, 84.31%), and the other manifestations were cognitive impairment (20/38, 53.63%), hemorrhagic stroke (7/38, 18.42%), migraine (9/38, 23.68%), and psychiatric disorders (8/38, 21.05%). Seizure was not detected in our patients. Skin biopsies were performed in 13 proband patients carrying *NOTCH3* variants, and GOM was detected in 11 proband patients (11/13, 84.62%).

**TABLE 1:** Summary of clinical features of 54 proband patients.

	Total (*n* = 54)	*NOTCH3* positive (*n* = 38)	*NOTCH3* negative (*n* = 16)	*p*-value^a^
Sex (M/F)	26/28	19/19	7/9	0.770
Age at onset (year, mean ± SD)	48.35 ± 9.24	47.18 ± 9.51	51.13 ± 8.17	0.154
Risk factor [number (%)]	22 (40.74%)	15 (39.47%)	7 (43.75%)	0.772
Symptoms [number (%)]				
Ischemic stroke	45 (83.33%)	32 (84.21%)	13 (81.25%)	>0.999
Hemorrhagic stroke	11 (20.37%)	7 (18.42%)	4 (25.00%)	0.714
Migraine	11 (20.37%)	9 (23.68%)	2 (12.50%)	0.474
Cognitive impairment	29 (53.70%)	20 (52.63%)	9 (56.25%)	>0.999
Psychiatric disorders	13 (24.07%)	8 (21.05%)	5 (31.25%)	0.493
WHM involvement [number (%)]				
External capsule	42 (77.78%)	33 (86.84%)	9 (56.25%)	**0.028**
Anterior temporal lobe	20 (37.04%)	17 (44.74%)	3 (18.75%)	0.122
GOM detected in skin biopsy (number (%))	11/16 (68.75%)	11/13 (84.62%)	0/3 (0.00%)	**0.018**

*^*a*^*NOTCH3* positive vs. *NOTCH3* negative. Bold values: *p* < 0.05.*

### Genetic Analysis

**FIGURE 1:**
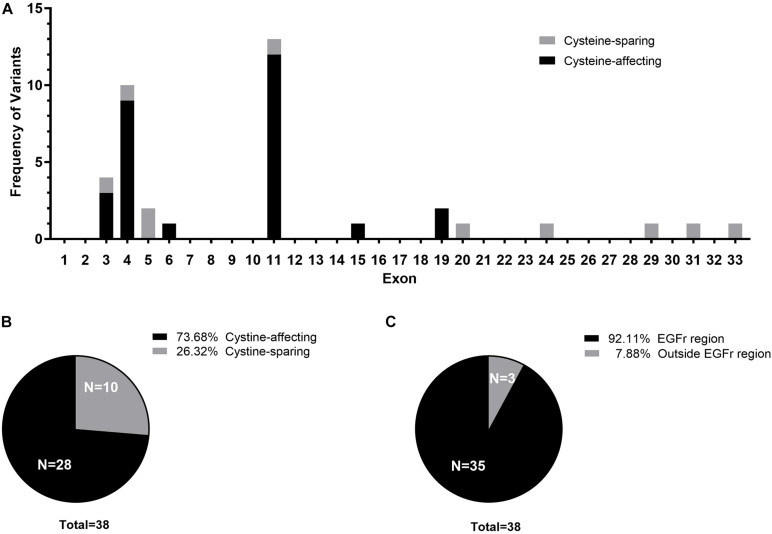
**(A)** Exon distribution of the cysteine-affecting and cystine-sparing *NOTCH3* variants identified in 38 patients. **(B)** Pie chart displaying the proportion of patients carrying cystine-affecting variants and cystine-sparing variants. **(C)** Pie chart displaying the proportion of patients carrying *NOTCH3* variants within EGFr region and outside EGFr region.

Twenty-three different *NOTCH3* heterozygous missense variants were identified from 38 (Table 2). Exon distribution of *NOTCH3* variants is shown in Figure 1A. Most variants were found in exon 11(13/38, 34.21%), followed by exon 4 (10/38, 26.32%), and exon 3 (4/38, 10.53%). Five different variants were found recurrently: R544C (9/38, 23.68%) was the most common recurrent variant, the rest four recurrent variants were R153C (3/38, 7.89%), R110C (2/38, 5.26%), V237M (2/38, 5.26%), and R607C (2/38, 5.26%).

**TABLE 2:** Spectrum of *NOTCH3* variants found in 38 Chinese CADASIL patients.

Nucleotide change	Amino acid change	Exon	EGFr domain	Cysteine affecting (Y/N)^b^	Frequency	Variants per exon [No. (%)]	Reported in CADASIL (Y/N)^c^	GOM deposit in biopsy^d^	ACMG^e^
224 G > C	R75P	3	1	N	1	10.53%	Y	NP*	LP
268 C > T	R90C	3	2	Y	1		Y	1/1	P
328 C > T	R110C	3	2	Y	2		Y	1/1	LP
397 C > T	R133C	4	3	Y	2	26.32%	Y	1/1	P
397 C > A	R133S	4	3	N	1		Y	1/1	LP
457 C > T	R153C	4	3	Y	3		Y	0/2	P
505 C > T	R169C	4	4	Y	1		Y	1/1	P
544 C > T	R182C	4	4	Y	2		Y	2/2	P
554 G > A	C185Y	4	4	Y	1		Y	1/1	P
709 G > A	V237M	5	5	N	2	5.26%	Y	NP	U
1013 G > C	C338S	6	8	Y	1	2.63%	Y	NP	P
1630 C > T	R544C	11	13/14	Y	9	34.21%	Y	2/2	LP
1759 C > T	R587C	11	15	Y	1		Y	NP	U
1819 C > T	R607C	11	15	Y	2		Y	NP	LP
1820 G > A	R607H	11	15	N	1		Y	1/1	LP
2299 C > T	R767C	15	19	Y	1	2.63%	Y	NP	U
3016 C > T	R1006C	19	26	Y	1	5.26%	Y	NP	LP
3062 A > G	Y1021C	19	26	Y	1		Y	NP	U
3299 G > A	R1100H	20	28	N	1	2.63%	Y	NP	U
4039 G > C	G1347R	24	34	N	1	2.63%	Y	NP*	U
5282 G > A	R1761H	29	N^*a*^	N	1	2.63%	Y	NP*	U
5764 G > T	V1922L	31	N^*a*^	N	1	2.63%	N	NP	U
6608 C > A	S2203Y	33	N^*a*^	N	1	2.63%	N	NP	U

^*a*^*Y*, in EGFr region; *N*, outside EGFr region. ^*b*^*Y*, cysteine affecting; *N*, cysteine sparing. ^*c*^*Y*, variant reported in CADASIL; *N*, novel variant in CADASIL. ^*d*^GOM-detected proband/studied proband; *NP*, skin biopsy not performed in present study; NP*, skin biopsy was not performed in present study but GOM deposit was reported in literature. ^*e*^Interpretation of variants based on consensus recommendation of the American College of Medical Genetics (ACMG): pathogenic (P), likely pathogenic (LP), and unknown significance (U).

Of the 23 *NOTCH3* variants, 14 variants from 28 patient (28/38, 73.68%) were cysteine-affecting variants located in the EGFr region. Nine variants from 10 patients (10/38, 26.32%) were cysteine sparing (Figure 1B). Among them, six variants (R75P, R133S, V237M, R607H, R1100H, and G1347R) were located within EGFr region, three variants (R1761H, V1922L, and S2203Y) were located outside EGFr region. Patients carrying variants outside EGFr region account for 7.88% of all patients (Figure 1C).

For patients carrying variants outside EGFr region, no potential pathogenic mutation were identified in several other genes known to be common causal of cerebral small-vessel disease or vascular dementia (*HTRA1*, *TREX1*, *COL4A1*, *COL4A2*, *GLA*, *APP*, and *ITM2B*) by targeted next-generation sequencing. None of the three variants outside EGFr region were detected in 400 age- and sex-matched Chinese controls.

### Family Information and Clinical Data of Patients Carrying Untypical Variants

**FIGURE 2:**
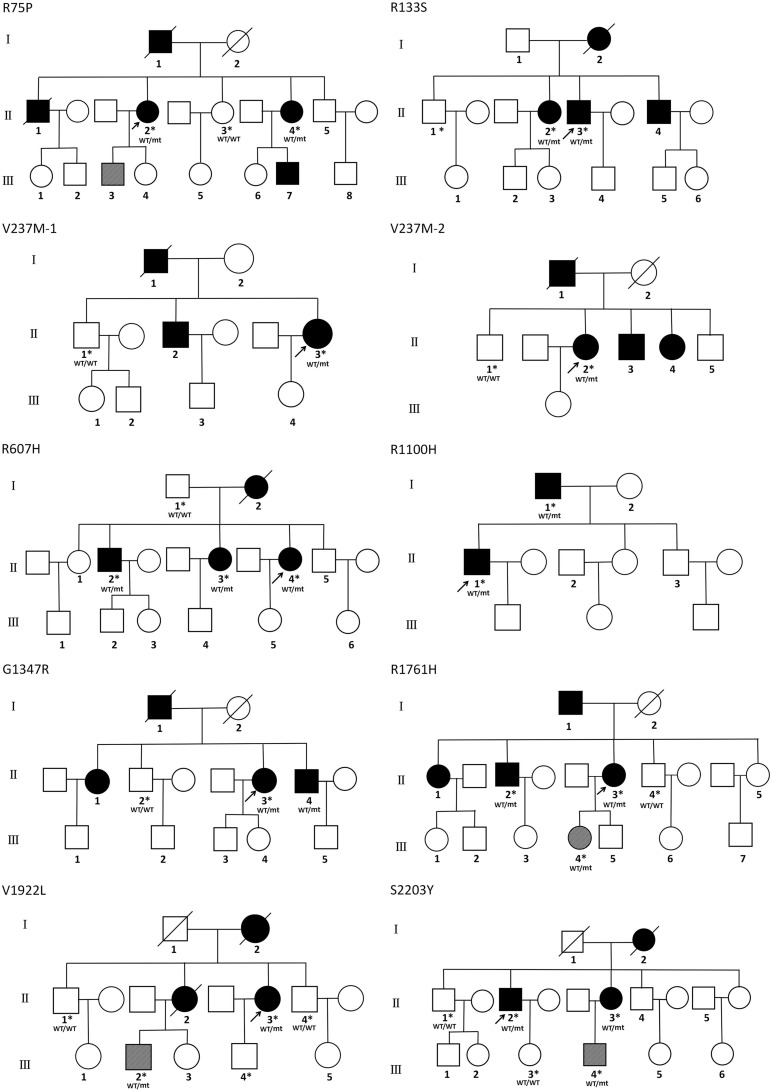
Family pedigrees of patients carrying untypical *NOTCH3* variants. The arrow points to the proband. Square and circles indicate males and females, respectively. A bar across the symbol means a decreased individual. Symptomatic family members are shown in black filled. Asymptomatic family members with MRI abnormalities are shown in stripe filled. Asterisks indicate gene-tested subjects. WT, wide type; mt, mutant variant.

**FIGURE 3:**
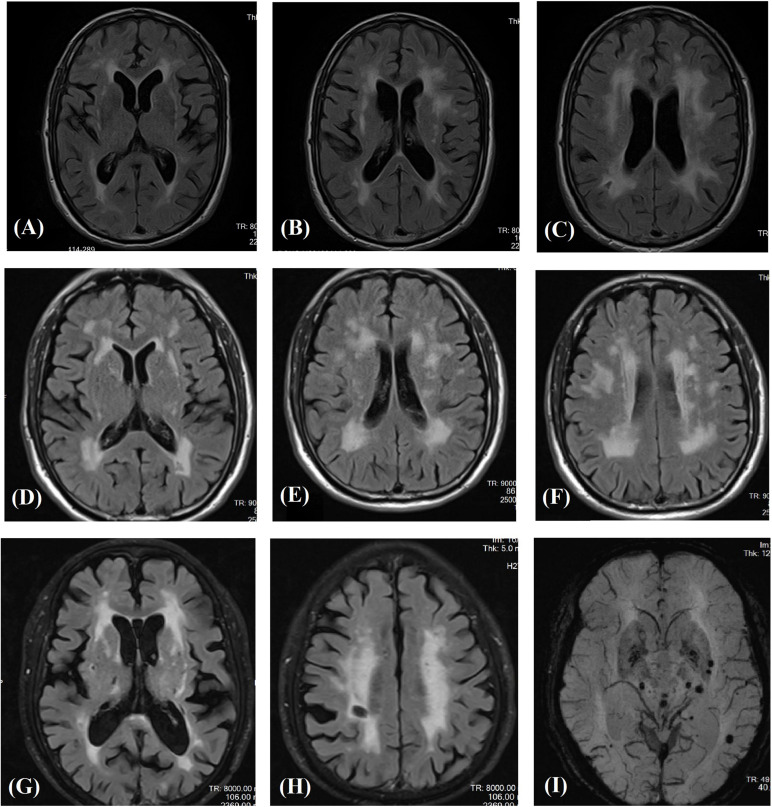
Magnetic resonance images in three patients carrying novel *NOTCH3* variants outside EGFr coding region. Fluid-attenuated inversion recovery images of the carrier of S2203Y **(A–C)**, V1922L **(D–F)**, and R1761H **(G,H)** showing multiple subcortical lacunar infarcts and characteristic diffuse leukoaraiosis with involvement of the external capsule. SWI images showed multiple microbleeds in basal ganglion and subcortical region of the carrier of R1761H **(I)**.

**FIGURE 4:**
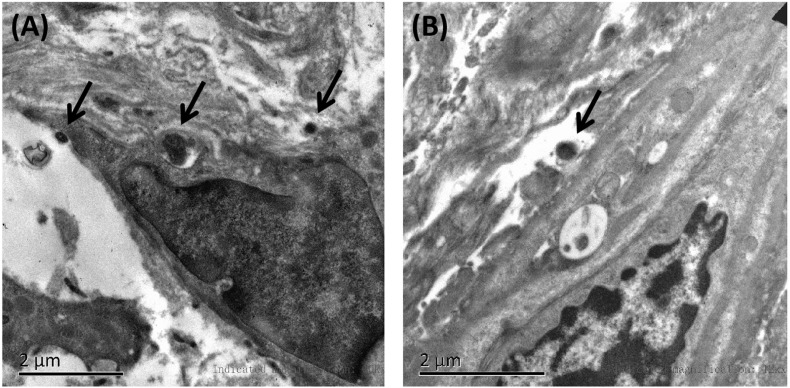
Representative images of skin biopsies. Electron microscopy of skin biopsies from the proband carrying R133S **(A)** and R607H **(B)**, showing deposits of GOM (black arrow).

Affected family members from 10 probands carrying 9 untypical variants (cysteine-sparing variants and variants outside EGFr region) are summarized in Table 3. Family pedigrees and family co-segregation analysis are shown in Figure 2. Detailed family information is shown in Supplementary Table 1. Representative neuroimaging of probands carrying variant outside EGFr region are presented in Figure 3, showing multiple subcortical lacunar infarcts, extensive WMH with the involvement of the external capsule, and multiple cerebral microbleeds. None of the proband carrying variant outside EGFr region showed WMH involved with anterior temporal lobe. GOM was detected in skin biopsies from patients carrying R133S and R607H (Figure 4).

**TABLE 3:** Clinical features of probands and affected family members carrying untypical *NOTCH3* variants.

Family	Patient ID^a^	Gender^b^	Age^c^	Onset age^d^	Clinical manifestation^e^	MRI features		Genotype^g^
						CMBs burden	WMH rating^*f*^	
R75P	I-1	M	67^#^	58	IS, CI	NP	NP	NP
	II-1	M	66^#^	51	IS, CI	NP	NP	NP
	**II-2**	F	74	72	IS, CI	1	3/5/5/5/0/1/3	R75P
	II-4	F	68	54 (43)	MA, IS, CI	1	2/6/5/3/1/1/1	R75P
	III-3	M	47	–	Asymptomatic	2	1/5/4/4/0/1/5	NP
	III-7	M	45	(38)	MA	1	0/2/3/4/2/0/5	NP
R133S	I-2	F	68^#^	50	IS, CI	NP	NP	NP
	II-2	F	54	51	IS	1	1/5/5/4/1/1/3	R133S
	**II-3**	M	50	42	IS, CI	2	2/5/6/5/4/3/6	R133S
	II-4	M	44	42	IS	NP	1/5/5/4/1/3/3	NP
	I-2	F	55^#^	55 (30)	MA, HS	NP	NP	NP
V237M-1	I-1	M	70^#^	51	HS, CI	NP	NP	NP
	II-2	M	56	42 (35)	MA. IS	2	5/4/4/4/3/1/3	NP
	**II-3**	F	54	51 (35)	MA, CI	2	1/6/6/6/0/1/3	V237M
V237M-2	I-1	M	70^#^	50	IS, CI	NP	NP	NP
	**II-2**	F	68	55	IS, CI	0	3/4/5/4/0/0/5	V237M
	II-3	M	65	53	IS, CI	2	1/5/5/5/0/1/2	NP
	II-4	F	63	50	IS	2	3/5/6/6/2/2/4	NP
R607H	I-2	F	55^#^	55 (30)	MA, HS	NP	NP	NP
	II-2	M	58	54	IS, CI	NP	2/5/6/6/5/1/1	R607H
	II-3	F	55	49	HS	1	1/5/5/5/3/1/1	R607H
	**II-4**	F	52	46 (38)	MA, IS	2	1/5/6/5/1/1/3	R607H
R1100H	I-1	M	74	50	IS, CI	NP	1/5/6/5/5/2/6	R1100H
	**II-1**	M	54	53	IS, CI	1	1/3/4/4/5/2/1	R1100H
G1347R	I-1	M	67^#^	50	IS, CI	NP	NP	NP
	II-1	F	63	52	IS, CI	1	1/5/5/4/0/1/5	G1347R
	**II-3**	F	58	51	IS, CI	2	0/6/4/6/3/0/3	G1347R
	II-4	M	55	48	IS	2	3/3/6/4/0/3/3	G1347R
R1761H	I-1	M	72	54	IS, CI	NP	1/5/6/6/0/3/4	R1761H
	II-1	F	58	54	IS, CI	1	0/4/4/3/3/1/0	R1761H
	II-2	M	52	45	CI, PD	NP	2/4/5/5/0/2/3	R1761H
	**II-3**	F	47	47	IS, PD	2	3/5/6/6/0/1/5	R1761H
	III-4	F	25	–	Asymptomatic	0	0/2/4/5/0/1/0	NP
V1922L	I-2	F	72^#^	55	IS, CI	NP	NP	NP
	II-2	F	60^#^	59	HS, CI	NP	NP	NP
	**II-3**	F	59	52	IS	1	0/5/4/4/0/1/5	V1922L
	III-2	M	38	–	Asymptomatic	1	1/3/3/3/1/3/5	V1922L
S2203Y	I-2	F	70^#^	57	IS, CI, MA	NP	NP	NP
	**II-2**	M	70	65	IS, HS, CI	2	3/5/6/5/0/3/5	S2203Y
	II-3	F	66	60	IS, CI	0	2/5/4/4/1/1/3	S2203Y
	III-4	M	40	–	Asymptomatic	1	3/5/5/5/1/2/3	S2203Y

*^*a*^Proband patients were boldfaced. ^*b*^*M*, male; *F*, female. ^*c*^Age at evaluation; ^#^deceased age. ^*d*^Onset age of migraine is shown in the bracket. ^*e*^*MA*, migraine; *CI*, cognitive impairment; *IS*, ischemia stroke; *HS*, hemorrhagic stroke; *PD*, psychiatric disorders. ^*f*^White matter hyperintensity rating by the modified Schelten’s scale. The score was recorded as infratentorial/periventricular/frontal/parietal/temporal/occipital/external capsule. *NP*, not performed. ^*g*^*NP*, not performed.*

### Pathogenicity Assessment and Classification of *NOTCH3* Variants

According to the consensus statement of *NOTCH3* variants causing CADASIL (Mancuso et al., 2020), all of the 14 cysteine-affecting *NOTCH3* variants in EGFr region were considered to be pathogenic variant because they fulfill the archetype of CADASIL-related mutation and had been reported in previous studies. *In silico* analysis, allele frequency, and pathological information of nine untypical variants are shown in Table 4. R75P, R133S, R607H, G1347H, and R1761H were considered to be pathogenic variant based on positive GOM deposit in skin biopsies in present (R133S and R607H) or previous studies (R75P, G1347H, and R1761H) (Nakamura et al., 2015; Ueda et al., 2015; Wang et al., 2020). V237M, R1100H, V1922L, and S2203Y were considered variant of unknown significance (VUS). The genotype-phenotype correlation analysis was based on the consensus statement of *NOTCH3* variants causing CADASIL (Mancuso et al., 2020).

**TABLE 4:** *In silico* analysis, allele frequency, and classification of untypical *NOTHC3* variants.

Variant	*In silico* analysis				Frequency in GnomAD	GOM deposit in biopsy	Classification^a^
	SIFT	PolyPhen-2	Mutation taster	GERP^++^			
R75P	0.132	0.987	0.961	5.13	<0.001	Positive*	PV
	Tolerated	Probably damaging	Polymorphism	Conserve			
R133S	0.662	0.033	1.000	5.02	<0.001	Positive	PV
	Tolerated	Benign	Disease causing	Conserve			
V237M	0.002	0.908	1.000	5.44	<0.001	NP	VUS
	Damaging	Probably damaging	Disease causing	Conserve			
R607H	0.576	0.006	0.977	4.51	<0.001	Positive	PV
	Tolerated	Benign	Disease causing	Conserve			
R1100H	0.120	0.056	1.000	5.08	<0.001	NP	VUS
	Tolerated	Benign	Disease causing	Conserve			
G1347R	0.008	0.978	1.000	4.48	<0.001	Positive*	PV
	Damaging	Probably damaging	Disease causing	Conserve			
R1761H	0.014	1.000	1.000	4.26	<0.001	Positive*	PV
	Damaging	Deleterious	Disease causing	Conserve			
V1922L	0.202	1.000	1.000	5.14	<0.001	NP	VUS
	Tolerated	Deleterious	Disease causing	Conserve			
S2203Y	0.037	0.978	0.698	5.14	<0.001	NP	VUS
	Damaging	Probably damaging	Disease causing	Conserve			

*GOM-positive reported in literature; *NP*, not performed. ^*a*^Classification following the consensus statement of *NOTCH3* variants causing CADASIL: pathogenic variant (PV) and variant of unknown significance (VUS).

### Genotype-Phenotype Correlation

**FIGURE 5:**
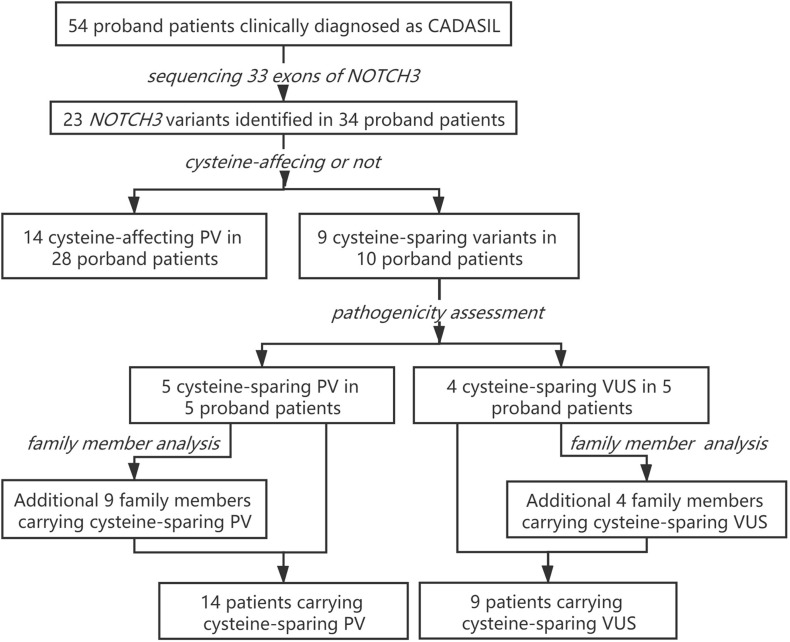
Patient enrollment and study work flow. PV, pathogenic variant; VUS, variant of unknown significance.

Clinical characteristics of patients carrying cystine-affecting pathogenic variants and cysteine-sparing pathogenic variants are shown in Table 5. The sex, prevalence of stroke risk factors, and clinical symptoms did not differ significantly between the two groups. We noticed that patients carrying cysteine-sparing pathogenic variants showed later symptom onset than patients carrying cysteine-affecting pathogenic variants, though the difference was insignificant (51.60 ± 11.84 vs. 44.96 ± 8.82, *p* = 0.150). The difference was significant when an additional nine genetically confirmed family members carrying cysteine-sparing pathogenic variants were included (51.36 ± 7.06 vs. 44.96 ± 8.82, *p* = 0.023). Though the prevalence of anterior temporal lobes involvement was not significantly different between two groups, the severity of temporal lobes involvement rated by modified Scheltens’s scale was lower in patients carrying cysteine-sparing pathogenic variants compared with those with cysteine-affecting pathogenic variants (1.50 ± 1.74 vs. 3.11 ± 2.32, *p* = 0.027). The patient enrollment and study work flow are shown in Figure 5.

**TABLE 5:** Comparison of clinical features between patients carrying cysteine-affecting and cysteine-sparing pathogenic *NOTHC3* variants.

	Proband patients carrying cysteine-affecting PV^a^ (*n* = 28)	Proband patients carrying cysteine-sparing PV^a^ (*n* = 5)	Family members carrying cysteine-sparing PV^a^ (*n* = 14)	*p*-value^b^	*p*-value^c^
Sex (M/F)	16/12	1/4	5/9	0.175	0.326
Age at onset (year, mean ± SD)	44.96 ± 8.82	51.60 ± 11.85	51.36 ± 7.06	0.150	**0.023**
Risk factor [number (%)]	11 (39.29%)	2 (40.00%)	6 (42.86%)	>0.999	>0.999
Symptoms [number (%)]					
Ischemic stroke	23 (82.14%)	5 (100.00%)	12 (85.71%)	0.569	>0.999
Hemorrhagic stroke	6 (21.43%)	0 (0.00%)	1 (7.14%)	0.556	0.392
Migraine	7 (25.00%)	1 (20.00%)	2 (14.29%)	>0.999	0.693
Cognitive impairment	13 (46.42%)	3 (60.00%)	9 (64.29%)	0.656	0.338
Psychiatric disorders	7 (25.00%)	1 (20.00%)	2 (14.29%)	>0.999	0.693
WHM involvement [number (%)]					
External capsule	24 (85.71%)	5 (100.00%)	13 (92.86%)	>0.999	0.650
Anterior temporal lobe	14 (50.00%)	2 (40.00%)	5 (28.57%)	>0.999	0.515
WMH rating^*d*^ (mean ± SD)					
Infratentorial	2.14 ± 1.76	1.80 ± 1.30	1.57 ± 1.02	0.682	0.269
Perivetricular	4.86 ± 0.89	5.20 ± 0.55	4.86 ± 0.77	0.411	>0.999
Frontal	5.21 ± 0.96	5.40 ± 0.89	5.29 ± 0.73	0.690	0.807
Parietal	4.86 ± 0.89	5.40 ± 0.55	4.79 ± 1.05	0.200	0.819
Temporal	3.11 ± 2.32	1.60 ± 1.82	1.50 ± 1.74	0.179	**0.027**
Occipital	1.68 ± 1.12	1.20 ± 1.10	1.43 ± 0.94	0.386	0.478
External capsule	2.75 ± 1.90	4.00 ± 1.41	2.93 ± 1.73	0.172	0.769
CMB burden [number (%)]					
0	3.00 (10.71%)	0 (0.00%)	0/11 (0.00%)	>0.999	0.545
1	12 (42.86%)	1 (20.00%)	6/11 (54.55%)	0.625	0.723
2	13 (46.43%)	4 (80.00%)	5/11 (45.45%)	0.335	>0.999
GOM detected in skin biopsy [number (%)]	9/11 (81.82%)	2/2 (100%)	2/2 (100.00%)	>0.999	>0.999

*^*a*^*PV*, pathogenic variant. ^*b*^Proband patients carrying cysteine-affecting PV vs. proband patients carrying cysteine-sparing PV. ^*c*^Proband patients carrying cysteine-affecting PV vs. genetically confirmed patients carrying cysteine-sparing PV. ^*d*^White matter hyperintensity rating by the modified Schelten’s scale.*

Clinical characteristics of patients carrying pathogenic variants and VUS are shown in Table 6. The onset age of patients carrying VUS was later than patients carrying pathogenic variants (55.29 ± 5.35 vs. 45.97 ± 9.44, *p* = 0.016).

**TABLE 6:** Comparison of clinical features between patients carrying pathogenic variants and VUS.

	Proband patients carrying PV^a^ (*n* = 33)	Proband patients carrying VUS^b^ (*n* = 5)	Family members carrying VUS included (*n* = 9)	*p*-value^c^	*p*-value^d^
Sex (M/F)	17/16	2/3	4/5	>0.999	>0.999
Age at onset (year, mean ± SD)	45.97 ± 9.44	55.40 ± 5.51	55.29 ± 5.35	**0.037**	**0.016**
Risk factor [number (%)]	11 (33.33%)	2 (40.00%)	4 (44.44%)	>0.999	0.698
Symptoms [number (%)]					
Ischemic stroke	23 (69.70%)	4 (80.00%)	6/7 (85.71%)	>0.999	0.650
Hemorrhagic stroke	6 (18.18%)	1 (20.00%)	1/7 (14.29%)	>0.999	>0.999
Migraine	7 (21.21%)	1 (20.00%)	1/7 (14.29%)	>0.999	>0.999
Cognitive impairment	16 (48.48%)	4 (80.00%)	6/7 (85.71%)	0.344	0.105
Psychiatric disorders	7 (21.21%)	0 (0.00%)	0/7 (0.00%)	0.561	0.317
WHM involvement [number (%)]					
External capsule	29 (87.88%)	4 (80.00%)	8 (88.89%)	0.527	>0.999
Anterior temporal lobe	16 (48.48%)	1 (20.00%)	2 (22.22%)	0.355	0.258
WMH rating^*e*^					
Infratentorial	2.09 ± 1.68	1.60 ± 1.34	1.67 ± 1.12	0.593	0.481
Perivetricular	4.91 ± 0.84	4.60 ± 1.14	4.56 ± 1.01	0.469	0.292
Frontal	5.24 ± 0.94	5.00 ± 1.00	4.78 ± 1.10	0.596	0.210
Parietal	4.94 ± 0.86	4.60 ± 0.89	4.44 ± 0.89	0.420	0.173
Temporal	2.88 ± 2.29	1.00 ± 2.24	1.44 ± 2.07	0.095	0.097
Occipital	1.61 ± 1.12	1.60 ± 1.34	1.67 ± 1.00	0.991	0.884
External capsule	2.94 ± 1.87	3.80 ± 1.79	4.00 ± 1.58	0.324	0.128
CMB burden [number (%)]					
0	3 (9.10%)	1 (20.00%)	2/8 (25.00%)	0.446	0.247
1	13 (39.39%)	2 (40.00%)	4/8 (50.00%)	>0.999	0.698
2	17 (51.52%)	2 (40.00%)	2/8 (25.00%)	>0.999	0.249
GOM detected in skin biopsy [number (%)]	9/11 (81.82%)	NP	NP	NP	NP

*^*a*^*PV*, pathogenic variant. ^*b*^*VUS*, variant of unknown significant. ^*c*^Proband patients carrying cysteine-affecting PV vs. proband patients carrying cysteine-sparing PV. ^*d*^Proband patients carrying cysteine-affecting PV vs. genetically confirmed patients carrying cysteine-sparing PV. ^*e*^White matter hyperintensity rating by the modified Schelten’s scale.*

## Discussion

Previous studies suggested that both ethnicity and founder effects contribute to the difference of *NOTCH3* spectrum in CADASIL patients from different populations. In most of the Caucasian population, *NOTCH3* mutations were frequently found in exons 2–6 (60–80%), particularly exon 4 (50–75%) (Joutel et al., 1997; Markus et al., 2002; Peters et al., 2005). Italian populations showed different spectrum with mutations in exon 4 representing only 20.6% of the total (Bianchi et al., 2015). In Asian populations, mutations were more frequently found in exon 11 (40–85%) than exon 4 (20–40%) in patients from Korea (Kim et al., 2014), Chinese mainland (Chen et al., 2017), and Chinese Taiwan (Liao et al., 2015). R544C in exon 11 is the most common mutation of *NOTCH3* in the Asian population. However, mutations in exon 11 are rare in Japan. In a retrospective study including 70 Japanese CADASIL patients with *NOTCH3* mutations, R544C was not detected (Ueda et al., 2015). In the present study, variants in exon 11 (32.43%) and exon 4 (27.03%) account for approximately half the total variants, suggesting exon 11 and exon 4 to be “hot regions” in our cohort. The remaining variants were distributed over the other *NOTCH3* exons. The “hot regions” of *NOTCH3* variants in our patients were similar to previous studies in Korea (Kim et al., 2014), Chinese mainland (Chen et al., 2017), and Chinese Taiwan (Liao et al., 2015). The difference is that we found a higher proportion of cysteine-sparing variants and variants outside the EGFr coding region.

The pathogenicity of cysteine-sparing mutation in EGFr region was controversial at first (Rutten et al., 2014), but accumulating evidences supported that cysteine-sparing mutation may be disease causing. Several cysteine-sparing mutations fulfill characteristics required to be considered potentially pathogenic (typical clinical features, diffuse WMH, thorough sequencing of EGFr coding exons, GOM deposition, and MAF < 0.001) (Muino et al., 2017). GOM deposits were observed in patients carrying cysteine-sparing mutations (Santa et al., 2003; Scheid et al., 2008; Brass et al., 2009; Wang et al., 2011; Wollenweber et al., 2015). *In vitro* studies showed that the aggregation behavior of cysteine-sparing mutants was similar to cysteine-affecting mutants (Wollenweber et al., 2015; Huang et al., 2020). We identified six cysteine-sparing variants in EGFr region, all of them has been reported in previous studies. Among them, R75P (Ueda et al., 2015) and G1347R (Nakamura et al., 2015) have been reported to be associated with positive GOM deposition. In present study, we detected GOM in proband patients carrying R133S and R607H, supporting R133S and R607H to be pathogenic variant of CADASIL. V237M has been reported in a Japanese family characterized by late-onset migraine, gait disturbance, and dementia with MRI changes suggestive of cerebral small vessel disease (Uchino et al., 2002). R1100H has been reported in a Chinese female characterized by stroke, memory impairment, migraine, and WMH involving temporal pole and external capsule (Qin et al., 2019). Unfortunately, none of the patients carrying V237M and R1100H in previous or present study performed a skin biopsy, the pathogenicity of V237M and R1100H is not sufficiently convincing and need further clarification.

Variant outside the EGFr region in CADASIL was first reported in 1997, when *NOTCH3* was characterized to be causative gene of CADASIL (Joutel et al., 1996). A heterozygous missense variant (c.5554 G > A) in exon 30 was identified in a Swiss family affected by recurrent stroke-like episodes, migraine, subcortical dementia, and positive GOM deposition. Up to date, there are six cases reported variants outside EGFr to be possible causal of CADASIL (summarized in Supplementary Table 2; Jung et al., 1995; Joutel et al., 1996; Fouillade et al., 2008; Bersano et al., 2012; Hung et al., 2018; Park et al., 2020). We identified three variants outside the EGFr coding region. All three patients have positive family history of migraine/stroke/dementia, suggesting an underlying genetic cause. Second, we excluded concurrence of other mutation in *NOTCH3* and common causative genes related to cerebral small-vessel disease or vascular dementia. Moreover, these variants were not found in 400 age- and sex-matched Chinese controls and were rare in general population with MAF < 0.001. *In silico* analysis predicted these variants to affect conserve amino-acid sequences and impair protein function. Based on the above reasons, we consider these three novel variants outside EGFr region might be causative of CADASIL phenotype.

It is hard to clarify the pathogenicity of variants in Notch3 intracellular domain based on current consensus that accumulation of Notch3 extracellular domain, not intracellular domain, is the pathogenic mechanism of CADASIL. However, variant outside EGFr associated with positive GOM has been reported in three patients carrying L1518M (Park et al., 2020), A1851T (Joutel et al., 1996), and R1761H (Wang et al., 2020), respectively. The mechanism on how intracellular mutant lead to deposition of extracellular domain is unclear. In addition, other mechanisms leading to vasculopathy different from accumulation of Notch3 intracellular domain cannot be ruled out. There were studies showing that some CADASIL-like symptoms may be related to dysregulated Notch3 signaling (Baron-Menguy et al., 2017; Coupland et al., 2018). Altered Notch3 signaling has been proved in variant outside EGFr region, for example, variant L1515P (Fouillade et al., 2008), suggesting that variant outside EGFr might lead to arteriopathy by dysregulating Notch3 signaling. The three variants outside EGFr region detected in our study were located in the intracellular domain of Notch3, which is responsible for downstream Notch signaling. Although effects of these variants on protein function are not known, several software programs predicted them to be deleterious. It is reasonable to postulate that these variants might change Notch signaling pathway to initiate CADASIL pathogenicity. More functional studies were needed to clarify the role of variants outside the EFGr region in notch signaling and pathogenesis of CADASIL.

Clinical characteristics of patients carrying cysteine-sparing variants have rarely been described in cohort. R75P, a recurrent variant in Asian CADASIL was reported to be associated with less temporal pole involvement (Ueda et al., 2015). A recent study in Korea CADASIL patients reported similar results that patients with cysteine-sparing *NOTCH3* variants showed less involvement of the anterior temporal lobes than those with cysteine-affecting *NOTCH3* variants (Kim et al., 2020). We found that patients carrying cysteine-sparing pathogenic variants showed later symptom onset and milder temporal lobe involvement than patient carrying cysteine-affecting pathogenic variants, suggesting that cysteine-sparing variants may be related to different phenotype from typical CADASIL.

We also observed that none of the patients carrying variants outside the EGFr region showed severe WMH in anterior temporal lobe. Similarly, R544C, a recurrent mutation in Asian CADASIL patients localizes not in EGFr but between EGFr 13 and 14, has been reported to be associated with later disease onset and less involvement of anterior temporal lobe (Liao et al., 2015). We speculate that variants outside the EGFr region may lead to a milder CADASIL phenotype. As little is known about *NOTCH3* variants outside the EFGr region, we advocate complete screening of *NOTCH3* locus to reveal the entire genetic spectrum, and this is not difficult to achieve in the context of high-throughput sequencing.

Some shortcomings need consideration. First, only two patients carrying untypical variants agreed to a biopsy. Second, large fragment insertion/deletion mutations of *NOTCH3* were not excluded. Third, because of the limited sample size, the genotype-phenotype correlation should be interpreted carefully and needs to be confirmed in larger cohorts.

## Conclusion

In conclusion, we exhibited a distinct spectrum of *NOTCH3* variants in Chinese CADASIL patients and intrigued a potential genotype-phenotype correlation. Our findings broadened spectrum of *NOTCH3* variants in CADASIL and draw attention to a potential role of *NOTCH3* variants outside the EGFr region. Complete screening of 33 *NOTCH3* coding exons is advocated to better understand CADASIL phenotype and genotype spectrum, and more functional investigation is needed to ascertain the role of new *NOTCH3* variants in Notch3 signaling and CADASIL pathogenesis.

## Data Availability Statement

The datasets presented in this study can be found in online repositories. The names of the repository/repositories and accession number(s) can be found below: https://doi.org/10.6084/m9.figshare.13585919.v2.

## Ethics Statement

The studies involving human participants were reviewed and approved by the Institutional Ethics Review Boards of Xiangya Hospital, Central South University. The patients/participants provided their written informed consent to participate in this study. Written informed consent was obtained from the individual(s) for the publication of any potentially identifiable images or data included in this article.

## Author Contributions

YH and LZ designed the research. YH, QS, YZ, and FY collected the clinical data. HT provided and summarized the neuroimages information. LY, YT, NX, and ML performed the genetic analysis and interpreted the data. XL performed the statistical analysis and visualized the data. YH and ZW wrote the manuscript. QS, HX, and LZ supervised the study and revised the manuscript. ZW and LZ provided the funding support. All authors contributed to the article and approved the submitted version.

## Conflict of Interest

The authors declare that the research was conducted in the absence of any commercial or financial relationships that could be construed as a potential conflict of interest.

## Footnotes

1 http://gnomad.broadinstitute.org
