# Supplementary material for: NOTCH3 Variants and Genotype-Phenotype Features in Chinese CADASIL Patients
Source: Front Genet. 2021 Jul 15;12:705284. doi: 10.3389/fgene.2021.705284 (PMC8320595; doi:10.3389/fgene.2021.705284)
Supplement: Supplementary file 1 [file Data_Sheet_1.docx]

Supplementary Material

# Supplementary Tables

Supplementary table 1 Family co-segregation analysis in patients carrying untypical variants

| **Family** | **Patient**  **ID** ^a^ | **Gender** ^b^ | **Age** ^c^ | **Onset**  **Age** ^d^ | **Clinical manifestation** ^e^ | **MRI features** ^f^ | | | | | | | | **Genotype** ^g^ |
| --- | --- | --- | --- | --- | --- | --- | --- | --- | --- | --- | --- | --- | --- | --- |
|  |  |  |  |  |  | **CMBs** | **WMH** | | | | | | |  |
|  |  |  |  |  |  |  | **IFT** | **PVH** | **Frontal** | **Parietal** | **Temporal** | **Occipital** | **External capsule** |  |
| R75P | I-1 | M | 67# | 58 | IS, CI | NP | NP | NP | NP | NP | NP | NP | NP | NP |
|  | I-2 | F | 78# | — | — | NP | NP | NP | NP | NP | NP | NP | NP | NP |
|  | II-1 | M | 66# | 51 | IS, CI | NP | NP | NP | NP | NP | NP | NP | NP | NP |
|  | **II-2** | F | 74 | 72 | IS, CI | 1 | 3 | 5 | 5 | 5 | 0 | 1 | 3 | R75P |
|  | II-3 | F | 70 | — | — | NP | 0 | 1 | 1 | 1 | 0 | 0 | 0 | WT |
|  | II-4 | F | 68 | 54(43) | MA, IS, CI | 1 | 2 | 6 | 5 | 3 | 1 | 1 | 1 | R75P |
|  | II-5 | F | 62 | — | — | NP | NP | NP | NP | NP | NP | NP | NP | NP |
|  | III-3 | M | 47 | — | Asymptomatic | 2 | 1 | 5 | 4 | 4 | 0 | 1 | 5 | NP |
|  | III-7 | M | 45 | (38) | MA | 1 | 0 | 2 | 3 | 4 | 2 | 0 | 5 | NP |
| R133S | I-1 | M | 75 | — | — | NP | NP | NP | NP | NP | NP | NP | NP | WT |
|  | I-2 | F | 68# | 50 | IS, CI | NP | NP | NP | NP | NP | NP | NP | NP | NP |
|  | II-1 | M | 56 | — | — | 0 | 0 | 1 | 1 | 1 | 0 | 0 | 0 | WT |
|  | II-2 | F | 54 | 51 | IS | 1 | 1 | 5 | 5 | 4 | 1 | 1 | 3 | R133S |
|  | **II-3** | M | 50 | 42 | IS, CI | 2 | 2 | 5 | 6 | 5 | 4 | 3 | 6 | R133S |
|  | II-4 | M | 44 | 42 | IS | NP | 1 | 5 | 5 | 4 | 1 | 3 | 3 | NP |
| V237M-1 | I-1 | M | 70# | 51 | HS, CI | NP | NP | NP | NP | NP | NP | NP | NP | NP |
|  | I-2 | F | 80 | — | — | NP | NP | NP | NP | NP | NP | NP | NP | NP |
|  | II-1 | M | 59 | — | — | 0 | 0 | 1 | 0 | 1 | 0 | 0 | 0 | NP |
|  | II-2 | M | 56 | 42(35) | MA. IS | 2 | 5 | 4 | 4 | 4 | 3 | 1 | 3 | NP |
|  | **II-3** | F | 54 | 51(35) | MA, CI | 2 | 1 | 6 | 6 | 6 | 0 | 1 | 3 | V237M |
| V237M-2 | I-1 | M | 70# | 50 | IS, CI | NP | NP | NP | NP | NP | NP | NP | NP | NP |
|  | I-2 | F | 84# | — | — | NP | NP | NP | NP | NP | NP | NP | NP | NP |
|  | II-1 | M | 70 | — | — | NP | NP | NP | NP | NP | NP | NP | NP | NP |
|  | **II-2** | F | 68 | 55 | IS, CI | 0 | 3 | 4 | 5 | 4 | 0 | 0 | 5 | V237M |
|  | II-3 | M | 65 | 53 | IS, CI | 2 | 1 | 5 | 5 | 5 | 0 | 1 | 2 | NP |
|  | II-4 | F | 63 | 50 | IS | 2 | 3 | 5 | 6 | 6 | 2 | 2 | 4 | NP |
|  | II-5 | M | 60 | — | — | NP | NP | NP | NP | NP | NP | NP | NP | NP |
| R607H | I-1 | M | 82 | — | — | NP | NP | NP | NP | NP | NP | NP | NP | NP |
|  | I-2 | F | 55# | 55（30） | MA, HS | NP | NP | NP | NP | NP | NP | NP | NP | NP |
|  | II-1 | F | 60 | — | — | NP | 0 | 1 | 1 | 0 | 0 | 0 | 0 | WT |
|  | II-2 | M | 58 | 54 | IS, CI | ND | 2 | 5 | 6 | 6 | 5 | 1 | 1 | R607H |
|  | II-3 | F | 55 | 49 | HS | 1 | 1 | 5 | 5 | 5 | 3 | 1 | 1 | R607H |
|  | **II-4** | F | 52 | 46（38） | MA, IS | 2 | 1 | 5 | 6 | 5 | 1 | 1 | 3 | R607H |
|  | II-5 | M | 49 | — | — | NP | 0 | 0 | 1 | 0 | 0 | 0 | 0 | NP |
| R1100H | I-1 | M | 74 | 50 | IS, CI | NP | 1 | 5 | 6 | 5 | 5 | 2 | 6 | R1100H |
|  | I-2 | F | 75 | — | — | NP | NP | NP | NP | NP | NP | NP | NP | WT |
|  | **II-1** | M | 54 | 53 | IS, CI | 1 | 1 | 3 | 4 | 4 | 5 | 3 | 1 | R1100H |
|  | II-2 | F | 52 | — | — | NP | 0 | 1 | 0 | 0 | 0 | 0 | 1 | WT |
|  | II-3 | M | 49 | — | — | NP | NP | NP | NP | NP | NP | NP | NP | NP |
| G1347R | I-1 | M | 67# | 50 | IS, CI | NP | NP | NP | NP | NP | NP | NP | NP | NP |
|  | I-2 | F | 81# | — | — | NP | NP | NP | NP | NP | NP | NP | NP | NP |
|  | II-1 | F | 63 | 52 | IS, CI | 1 | 1 | 5 | 5 | 4 | 0 | 1 | 5 | G1347R |
|  | II-2 | M | 60 | — | — | NP | 0 | 2 | 0 | 0 | 0 | 0 | 0 | WT |
|  | **II-3** | F | 58 | 51 | IS, CI | 2 | 0 | 6 | 4 | 6 | 3 | 0 | 3 | G1347R |
|  | II-4 | M | 55 | 48 | IS | 2 | 3 | 3 | 6 | 4 | 0 | 3 | 3 | G1347R |
| R1761H | I-1 | M | 72 | 54 | IS, CI | NP | 1 | 5 | 6 | 6 | 0 | 3 | 4 | R1761H |
|  | I-2 | F | 55# | — | — | — | — | NP | NP | NP | NP | NP | NP | NP |
|  | II-1 | F | 58 | 54 | IS, CI | 1 | 0 | 4 | 4 | 3 | 3 | 1 | 0 | R1761H |
|  | II-2 | M | 52 | 45 | CI, PD | NP | 2 | 4 | 5 | 5 | 0 | 2 | 3 | R1761H |
|  | **II-3** | F | 47 | 47 | IS, PD | 2 | 3 | 5 | 6 | 6 | 0 | 1 | 5 | R1761H |
|  | II-4 | M | 45 | — | — | 0 | 0 | 0 | 1 | 0 | 0 | 0 | 0 | WT |
|  | II-5 | F | 42 | — | — | NP | NP | NP | NP | NP | NP | NP | NP | NP |
|  | III-4 | F | 25 | — | Asymptomatic | 0 | 0 | 2 | 4 | 5 | 0 | 1 | 0 | NP |
| V1922L | I-1 | M | 82# | — | — | NP | NP | NP | NP | NP | NP | NP | NP | NP |
|  | I-2 | F | 72# | 55 | IS, CI | NP | NP | NP | NP | NP | NP | NP | NP | NP |
|  | II-1 | M | 65 | — | — | 0 | 1 | 1 | 0 | 0 | 0 | 0 | 0 | WT |
|  | II-2 | F | 60# | 59 | HS, CI | NP | NP | NP | NP | NP | NP | NP | NP | NP |
|  | **II-3** | F | 59 | 52 | IS | 1 | 0 | 5 | 4 | 4 | 0 | 1 | 5 | V1922L |
|  | II-4 | M | 54 | — | — | 0 | 0 | 0 | 0 | 0 | 0 | 0 | 0 | WT |
|  | III-2 | M | 38 | — | Asymptomatic | 1 | 1 | 3 | 3 | 3 | 1 | 3 | 5 | V1922L |
| S2203Y | I-1 | M | 77# | — | — | NP | NP | NP | NP | NP | NP | NP | NP | NP |
|  | I-2 | F | 70# | 57 | IS, CI, MA | NP | NP | NP | NP | NP | NP | NP | NP | NP |
|  | II-1 | M | 73 | — | — | NP | 1 | 2 | 1 | 1 | 0 | 0 | 0 | WT |
|  | **II-2** | M | 70 | 65 | IS, HS, CI | 2 | 3 | 5 | 6 | 5 | 0 | 3 | 5 | S2203Y |
|  | II-3 | F | 66 | 60 | IS, CI | 0 | 2 | 5 | 4 | 4 | 1 | 1 | 3 | S2203Y |
|  | II-4 | M | 65 | — | — | NP | NP | NP | NP | NP | NP | NP | NP | NP |
|  | II-5 | F | 62 | — | — | NP | NP | NP | NP | NP | NP | NP | NP | NP |
|  | III-4 | M | 40 | — | Asymptomatic | 1 | 3 | 5 | 5 | 5 | 1 | 2 | 3 | S2203Y |

^a^: Proband patients were shown in bold fonts.

^b^: M = male, F = female.

^c^: Age at evaluation, # = deceased age.

^d^: onset age of migraine was shown in the bracket.

^e^: MA = migraine, CI = cognitive impairment, IS = ischaemia stroke, HS = hemorrhagic stroke, PD = psychiatric disorders.

^f^: CMB = cerebral microbleeds, WMH = white matter hyperintensity, IFT = infratentorial, PVH = periventricular hyperintensity.

^g^: NP = not performed.

Supplementary table 2 Summary of reported *NOTCH3* variants outside EGFr region

| **Nucleotide change** | **Amino acid change** | **Exon** | **Population** | **Families and patients** | **Clinical symptom** | **Onset age (year)** | **MRI findings** | **Biopsy** | **Frequency in control cohort** | **MAF in**  **ExAC/1000G** | **Reference** |
| --- | --- | --- | --- | --- | --- | --- | --- | --- | --- | --- | --- |
| 4544 T>C | L1515P | 25 | French | **1A** | Recurrent stroke, migraine | 35 | Bilateral WMH, dialated perivascular spaces, EC(+), AT(-) | Negative | 0/210 | 0/0 | (Fouillade et al., 2008) |
| 4552C > A | L1518M | 25 | Korean | **2A** | Progressive gait difficulties | 50 | Bilateral lesions in anterior temporal, external capsular, and periventricular region | Positive | NP | 0.00201/  0.0058 | (Park et al., 2020) |
| 5282 G>A | R1761H | 29 | Chinese | 3A | Mood disturbance, cognitive disorder, stroke | 40 | Severe leukoencephalopathy subcortical infarction, CMBs | Positive | 0/200 | ＜0.001/0 | (Wang et al., 2020) |
|  |  |  |  | 3B | Dizziness,stoke | 72？ | Leukoencephalopathy, subcortical infarction |  |  |  |  |
|  |  |  |  | 3C | TIA | 43 | NP |  |  |  |  |
| 5284G>A | V1762M | 29 | Italian | **4A** | Migraine, psychiatric disorders leukoencephalopathy, vasovagal syncope | 49 | Symmetric WMH, dialated perivascular spaces, EC(+), AT(-) | Negative | 0/200 | <0.0001/0 | (Bersano et al., 2012) |
|  |  |  |  | 4B | Asymptomatic | 88 | NP |  |  |  |  |
|  |  |  |  | 4C | Asymptomatic | 60 | NP |  |  |  |  |
|  |  |  |  | 4D | Psychiatric disorders | 55 | NP |  |  |  |  |
| 5554 G>A | A1852T | 30 | Swiss | 5A | Recurrent stroke episodes, progressive dementia | 45 | NP | Positive | 0/200 | 0/0 | (Jung et al., 1995; Joutel et al., 1996) |
|  |  |  |  | 5B | Migraine at age 40, progressive dementia at age 50, recurrent stroke episodes at age 55 | 40 | CT: bilateral hypoindensities in the white matter, leukoaraiosis |  |  |  |  |
|  |  |  |  | 5C | Transient stoke-like episode at age 53, progressive dementia at age 59, stroke at age 62 | 53 | CT:bilateral hypoindensities in the white matter, leukoaraiosis |  |  |  |  |
|  |  |  |  | 5D | Episode of major depression at age 40, transient stoke-like episode at age 52, progressive dementia and recurrent stroke at age 54 | 40 | CT:bilateral hypoindensities in the white matter, leukoaraiosis |  |  |  |  |
|  |  |  |  | **5E** | Migraine, transient stoke-like episode | 33 | MRI: bilateral WHM and leukoencephalopathy |  |  |  |  |
|  |  |  |  | 5F | Migraine, transient stoke-like episode at age 37 | 35 | MRI: bilateral WHM and leukoencephalopathy |  |  |  |  |
|  |  |  |  | 5G | Stroke-like episodes, mild neuropsychological deficits | 38 | MRI: bilateral WHM and leukoencephalopathy |  |  |  |  |
| c.5903_5904 insATAA | D1969* | 32 | Chinese HongKong | **6A** | Progressive memory decline and leukoencephalopathy | 52 | Symmetric WMH, EC(+), AT(+) | NP | NP | 0/0 | (Hung et al., 2018) |
|  |  |  |  | 6B | Dementia and stroke | 50 | NP |  |  |  |  |
|  |  |  |  | 6C | Memory decline | 47 | NP |  |  |  |  |

WMH = white matter hyperintensity, CMBs = cerebral microbleeds, EC = external capsule involvement, AT = anterior temporal involvement, NP = not performed.

Index patient is boldfaced.

MAF = minor allele frequency.

**References**

Bersano, A., Ranieri, M., Ciammola, A., Cinnante, C., Lanfranconi, S., Dotti, M.T., et al. (2012). Considerations on a mutation in the NOTCH3 gene sparing a cysteine residue: a rare polymorphism rather than a CADASIL variant. *Funct Neurol* 27(4)**,** 247-252.

Fouillade, C., Chabriat, H., Riant, F., Mine, M., Arnoud, M., Magy, L., et al. (2008). Activating NOTCH3 mutation in a patient with small-vessel-disease of the brain. *Hum Mutat* 29(3)**,** 452. doi: 10.1002/humu.9527.

Hung, L.Y., Ling, T.K., Lau, N.K.C., Cheung, W.L., Chong, Y.K., Sheng, B., et al. (2018). Genetic diagnosis of CADASIL in three Hong Kong Chinese patients: A novel mutation within the intracellular domain of NOTCH3. *J Clin Neurosci* 56**,** 95-100. doi: 10.1016/j.jocn.2018.06.050.

Joutel, A., Corpechot, C., Ducros, A., Vahedi, K., Chabriat, H., Mouton, P., et al. (1996). Notch3 mutations in CADASIL, a hereditary adult-onset condition causing stroke and dementia. *Nature* 383(6602)**,** 707-710. doi: 10.1038/383707a0.

Jung, H.H., Bassetti, C., Tournier-Lasserve, E., Vahedi, K., Arnaboldi, M., Arifi, V.B., et al. (1995). Cerebral autosomal dominant arteriopathy with subcortical infarcts and leukoencephalopathy: a clinicopathological and genetic study of a Swiss family. *J Neurol Neurosurg Psychiatry* 59(2)**,** 138-143. doi: 10.1136/jnnp.59.2.138.

Park, D.G., Min, J.H., Sohn, S.H., Sohn, Y.B., and Yoon, J.H. (2020). Ataxia Associated with CADASIL: a Pathology-Confirmed Case Report and Literature Review. *Cerebellum* 19(6)**,** 907-910. doi: 10.1007/s12311-020-01173-z.

Wang, W., Ren, Z., Shi, Y., and Zhang, J. (2020). A Novel Mutation Outside of the EGFr Encoding Exons of NOTCH3 Gene in a Chinese with CADASIL. *J Stroke Cerebrovasc Dis* 29(12)**,** 105410. doi: 10.1016/j.jstrokecerebrovasdis.2020.105410.
